# Single-Molecule RNA Sequencing Reveals IFNγ-Induced Differential Expression of Immune Escape Genes in Merkel Cell Polyomavirus–Positive MCC Cell Lines

**DOI:** 10.3389/fmicb.2021.785662

**Published:** 2021-12-22

**Authors:** Tatjana Sauerer, Christopher Lischer, Adrian Weich, Carola Berking, Julio Vera, Jan Dörrie

**Affiliations:** ^1^RNA-based Immunotherapy, Hautklinik, Comprehensive Cancer Center Erlangen European Metropolitan Area of Nuremberg, Deutsches Zentrum Immuntherapie, Universitätsklinikum Erlangen, Friedrich-Alexander-Universität Erlangen-Nürnberg, Erlangen, Germany; ^2^Systems Tumor Immunology, Hautklinik, Universitätsklinikum Erlangen, Friedrich-Alexander-Universität Erlangen-Nürnberg, Comprehensive Cancer Center Erlangen European Metropolitan Area of Nuremberg, Deutsches Zentrum Immuntherapie, Erlangen, Germany; ^3^Hautklinik, Universitätsklinikum Erlangen, Friedrich-Alexander-Universität Erlangen-Nürnberg, Comprehensive Cancer Center Erlangen European Metropolitan Area of Nuremberg, Deutsches Zentrum Immuntherapie, Erlangen, Germany

**Keywords:** Merkel cell carcinoma (MCC), third generation sequencing, Interferon gamma (IFNγ), immune escape, transcriptome

## Abstract

Merkel cell carcinoma (MCC) is a rare and highly aggressive cancer, which is mainly caused by genomic integration of the Merkel cell polyomavirus and subsequent expression of a truncated form of its large T antigen. The resulting primary tumor is known to be immunogenic and under constant pressure to escape immune surveillance. Because interferon gamma (IFNγ), a key player of immune response, is secreted by many immune effector cells and has been shown to exert both anti-tumoral and pro-tumoral effects, we studied the transcriptomic response of MCC cells to IFNγ. In particular, immune modulatory effects that may help the tumor evade immune surveillance were of high interest to our investigation. The effect of IFNγ treatment on the transcriptomic program of three MCC cell lines (WaGa, MKL-1, and MKL-2) was analyzed using single-molecule sequencing *via* the Oxford Nanopore platform. A significant differential expression of several genes was detected across all three cell lines. Subsequent pathway analysis and manual annotation showed a clear upregulation of genes involved in the immune escape of tumor due to IFNγ treatment. The analysis of selected genes on protein level underlined our sequencing results. These findings contribute to a better understanding of immune escape of MCC and may help in clinical treatment of MCC patients. Furthermore, we demonstrate that single-molecule sequencing can be used to assess characteristics of large eukaryotic transcriptomes and thus contribute to a broader access to sequencing data in the community due to its low cost of entry.

## Introduction

Merkel cell carcinoma (MCC) is a rare and frequently metastatic neuroendocrine skin cancer with increasing incidence and high mortality (Fitzgerald et al., 2015; Paulson et al., 2018). Risk factors are increased age, UV light exposure, immunosuppression, and infection with the human Merkel cell polyomavirus (MCPyV; Heath et al., 2008). About 80% of all MCC tumors contain a mutated MCPyV genome that expresses viral oncoproteins (T antigens) (Feng H. et al., 2008; Houben et al., 2010). For malignant transformation, the MCPyV DNA is clonally integrated into the MCC genome in a replication-deficient form and expresses a truncated oncoprotein, called the large T (LT) antigen (Shuda et al., 2008). As this protein contains several tumor suppressors targeting motifs such as the retinoblastoma binding site, it promotes cell cycle progression that leads to uncontrolled cell proliferation (Borchert et al., 2014; Hesbacher et al., 2016). MCC is also characterized by an increased expression of programmed cell death protein 1 (PD-1), which allows the tumor cells to escape the control of the immune system (Afanasiev et al., 2013). Since 2018, checkpoint inhibitors (targeting PD-1 and its ligand PD-L1) have been approved by the Food and Drug Administration for the treatment of advanced MCC and show impressive clinical results, albeit not all patients respond to this treatment (Kaufman et al., 2016; Nghiem et al., 2016). It is suggested that MCC, as an immunogenic tumor, exerts different strategies of immune escape because it is capable of disturbing cellular immune responses by upregulation of PD-1 and PD-L1 and reducing the human leukocyte antigen (HLA) class I expression, leading to decreased or absent T cell infiltration (Ritter et al., 2017). The exact mechanism of immune escape is of high clinical relevance especially regarding immunotherapy resistance; however, it is not fully understood yet.

Interferons (IFNs) are suggested to beneficially influence antiviral immune responses and possess anti-polyomavirus (Co et al., 2007; Willmes et al., 2012) and anti-MCC activity (Krasagakis et al., 2008; Willmes et al., 2012), which makes them an interesting study subject in MCC. The type II IFN gamma (IFNγ) is of special interest, as it is well known for its ambiguous effects: Both anti-tumoral and pro-tumoral functions of IFNγ have been reported.

A closer investigation of the anti-tumoral effects shows that IFNγ is produced by activated lymphocytes including CD4^+^ and CD8^+^ T cells (Kasahara et al., 1983; Matsushita et al., 2015), gamma delta T cells (Gao et al., 2003), natural killer (NK) cells (Cooper et al., 2001; Yu et al., 2006), and NK T cells (Leite-De-Moraes et al., 1998). IFNγ plays a key role in modulating the immune response toward these effector cells in the tumor microenvironment. Its anti-tumor activity has been shown for fibrosarcoma and melanoma by the induction of HLA-I and signal transducer and activator of transcription 1 (STAT1)–associated cyclin-dependent kinase, leading to apoptosis of the tumor cells and recognition by the immune system (Ikeda et al., 2002; Gao et al., 2016). For MCC cell lines, it was shown that incubation with IFNγ slightly inhibited cell viability, reduced the expression of the MCPyV LT antigen (Willmes et al., 2012), and increased HLA-I expression (Paulson et al., 2014).

In contrast, pro-tumoral effects of IFNγ have been observed in colon carcinoma and melanoma, leading to a loss of IFNγ signaling sensitivity, reduced antigen expression (Beatty and Paterson, 2001), and induction of key immune suppressive molecules such as indoleamine-2,3-dioxygenase (IDO1) (Spranger et al., 2013) and immune checkpoint proteins (Benci et al., 2016). Hence, the same IFNγ signaling processes that promote anti-tumor immunity can achieve the opposite effect by inducing feedback inhibition and tumor immune escape (Bald et al., 2014).

To better understand the biology of a tumor and improve targeted therapies, high-throughput sequencing has been highly beneficial. The analysis of gene expression profiles of melanoma patients with checkpoint inhibitor treatment detected IFNγ-related mRNA profiles, which predicted clinical response to PD-1 treatment, underlining the huge clinical benefit of transcriptome sequencing studies (Ayers et al., 2017). Nanopore sequencing by Oxford Nanopore Technologies (ONT) belongs to the third-generation sequencing methods and enables real-time DNA and RNA sequencing using small nanopores (Jain et al., 2015, 2016). In this system, the DNA or RNA strand is guided through the nanopore creating nucleotide sequence-specific disturbance of the flow of an electrical current (Ip et al., 2015). In this process, up to 2 × 10^6^ bases can be read continuously (Payne et al., 2019). Nanopore sequencing shows increasing popularity in the field of cancer biology (Euskirchen et al., 2017) and virology (Quick et al., 2016). It is characterized by relatively low establishment and maintenance costs as well as rapid sequencing and real-time data acquisition for further analysis.

As the comprehensive investigation of the transcriptomic response of MCC to IFNγ is of high interest, we used the Nanopore sequencing platform to study differentially expressed genes in MCC. We hypothesized that IFNγ stimulation of MCC cell lines would lead to transcriptomic alterations that elucidate the ambiguous pro- and anti-tumoral activity of this cytokine and hence examined the effect of IFNγ on the transcriptomes of three MCPyV-positive cell lines (WaGa, MKL-1, and MKL-2). Our results confirm the Nanopore sequencing technology as a suitable tool to study these alterations and show a clear upregulation of genes involved in tumor immune escape mechanisms, suggesting that these factors could contribute to the immune escape of MCC cells.

## Materials and Methods

### Cell Culture

The MCC cell lines WaGa (RRID:CVCL_E998) (Houben et al., 2010), MKL-1(RRID:CVCL_EQ70) (Rosen et al., 1987), and MKL-2 (RRID:CVCL_D027) (Martin et al., 1991) were grown in Roswell Park Memorial Institute 1640 medium (Lonza, Verviers, Belgium) supplemented with 10% fetal bovine serum (FBS) (Merck KGaA, Darmstadt, Germany), 2 mM L-glutamine (Lonza), gentamycin (20 mg/L; Lonza), 10 mM hydroxyethyl piperazineethanesulfonic acid (HEPES; PAA Labortechnik, Pasching, Austria), and 20 mM beta-mercaptoethanol (Gibco) and maintained at 37°C in 5% CO_2_. All cells were kindly provided by Prof. Jürgen Becker (DKTK, Essen, Germany), and mycoplasma testing was performed immediately after arrival of the cells. Cells were frozen and then thawed for further experiments, whereby they were cultured for 1 to 3 weeks before the experiments were performed.

### Interferon Gamma Incubation and RNA Isolation of Cell Lines

For RNA extraction, 5 × 10^6^ cells of each cell lines were seeded at six-well plates, and IFNγ (3,000 U/ml; PeproTech, Hamburg, Germany) was added in each cell suspension. Cells incubated without IFNγ served as control. After 24 h of incubation, cell pellets were stored at −80°C until further analysis. For each cell line, the IFNγ incubation was performed three times on different days to obtain three replicates and exclude any daily fluctuation.

From frozen cell pellets, RNA isolation was performed using the AllPrep DNA/RNA micro kit (Qiagen, Venlo, Netherlands). RNA concentration was measured *via* Nanodrop and stored at −20°C until library preparation.

### Flow Cytometry Detection of Intracellular and Extracellular Markers

For antibody staining, 1 × 10^6^ cells of each MCC cell line were cultured in a six-well plate in the presence or absence of IFNγ (3,000 U/ml) for 72 h. Cells were washed with fluorescence-activated cell sorting (FACS) buffer (dulbecco’s phosphate-buffered saline with 1% FBS and 0.2% sodium azide) and stained for the following markers: STAT1α/β, Indoleamine-2,3-dioxygenase (IDO), C-X-C motif chemokine 10 (CXCL10), PD-L1, bone marrow stromal antigen 2 (BST2), HLA class II histocompatibility antigen gamma chain (CD74), and HLA A, B, and C (HLA-ABC) with the following monoclonal antibodies: anti-human STAT1 (phycoerythine; PE, clone REA272, Miltenyi Biotec), anti-IDO1 (PE, clone eyedio, Invitrogen), anti-human CXCL10 (PE, clone REA334, Miltenyi Biotec), anti-CD274 (Pe-Cy7, clone MIH1, BD Biosciences), anti-human BST2 (PE, clone REA202, Miltenyi Biotec), anti-CD74 (PE, clone 5-329, Miltenyi Biotec), and anti–HLA-ABC (fluorescein-5-isothiocyanat, BD Biosciences). STAT1, IDO, CXCL10, and CD74 were stained intracellularly using the Cytofix/Cytoperm™ Fixation/Permeabilization Solution Kit (Thermo Fisher Scientific, Waltham, MA, United States). PD-L1, BST2, HLA-ABC, and CD74 were stained as surface molecule. Stained cells were analyzed using a FACSCanto II flow cytometer (BD Biosciences) and FlowJo software, version 10.5.3 (BD Biosciences). Geometric mean of the mean fluorescence intensity was compared between ± IFNγ-treated cell lines.

### Preparation of Cell Lysates and Immunoblotting of the Merkel Cell Polyomavirus–Large T Antigen

Cell lysates were prepared out of 1 × 10^6^ cells for each cell line (WaGa, MKL-1, and MKL-2), incubated with or without IFNγ (3,000 U/ml) for 72 h. Cell pellets were frozen at −80°C, thawed, and resuspended in 35 μl of lysis buffer [10% glycerol, 2 mM ethylenediaminetetraacetic acid (EDTA), 137 mM NaCl, 50 mM Tris (pH 8.0) (all from Carl Roth GmbH, Germany), 0.5% sodium deoxycholate (Merck KGaA, Darmstadt, Germany)] freshly supplemented with 2 mM phenylmethylsulfonyl fluoride (Carl Roth GmbH, Germany), 2 mM sodium orthovanadate (Merck KGaA, Darmstadt, Germany), 20 mM sodium fluoride (Merck KGaA, Darmstadt, Germany), 0.1 M MgCl_2_ (Carl Roth GmbH, Germany), and 0.1 μl of benzonase (250 U/μl, Merck KGaA, Darmstadt, Germany) and lysed on ice for 20 min. Cells were then centrifuged at 13,500 × *g* at 4°C for 20 min, supernatants were harvested, and the protein concentration in each lysate was determined using Bradford protein determination. Protein lysates were mixed with 4× Roti-load 1 (Carl Roth GmbH, Germany), followed by a denaturation step at 95°C for 10 min.

Per lane, 20 μg of protein lysate of the three MCC cell lines were loaded onto 12% sodium dodecylsulfate (SDS) polyacrylamide gels and separated using SDS-polyacrylamide gel electrophoresis (PAGE). Afterward, proteins were transferred onto a nitrocellulose membrane by semi-dry blot transfer. After blocking the membrane in 1× Roti-block (Carl Roth GmbH, Germany) for 1 h at room temperature, the membrane was incubated with anti-MCPyV LT antigen (clone CM2B4, Merck KGaA) diluted 1:1,000 in 1× Roti-block overnight at 4°C and with the horseradish peroxidase-conjugated secondary antibody (polyclonal anti-mouse immunoglobulin G; Cell Signaling, 1:2,500) for 1 h. The antibodies were detected *via* enhanced chemoluminescence (ECL) using Amersham ECL Prime Western blotting detection reagent (GE Healthcare, Solingen, Germany) and documented and quantified *via* the Amersham ImageQuant system (GE Healthcare). glyceraldehyde-3-phosphate dehydrogenase (GAPDH) (anti-GAPDH mouse monoclonal Ab, clone 6C5, Merck KGaA, Darmstadt, Germany) was used as a loading control.

### Library Preparation and Nanopore Sequencing

Reagents and equipment for preparation and sequencing were obtained from ONT, Oxford, United Kingdom, unless stated otherwise. Library preparation was performed according to the instructions of the manufacturer using the PCR-cDNA Barcoding Kit (SQK-PCB109, ONT). Polyadenylated mRNA was reversely transcribed into cDNA and fused to barcodes (BCs) to allow sample-specific attribution. Of each RNA sample, 50 ng were used for library preparation. In total, six samples were loaded onto one flow cell with three biological replicates for each condition. Assigned BCs used were 1 through 6 for MKL-1 and 1, 2, 4, 5, 6, and 7 for both MKL-2 and WaGa.

Libraries were run for 72 h according to the guidelines of the manufacturer using the MK1B-MinION sequencer and R9.4 flow cells (FLO-MIN106, ONT). For each cell line, six samples (three replicates incubated with IFNγ and three replicates incubated without IFNγ) were loaded on one flow cell. Sequencing run monitoring and real-time data acquisition were performed using the MinKNOW software suite (v19.2.5). After real-time data were gathered, a high-accuracy base calling was performed using the CPU version of Guppy (v.4.2.2) deployed on a dedicated server, using the following command:

guppy_basecaller -r -i “fast5” -s
“sample” —num-callers \
1 –trim-barcodes –cpu_threads_per_caller 64 \
–c dna_r9.4.1_450bps_hac.cfg \
––barcode-kits SQK-PCB109 -q 0

### Data Processing

After a high-accuracy base calling was performed, generated FASTQ files were aggregated per sample and filtered for average Phred score quality above 7 (Q>7). In addition, primary quality assessment was done with the package pycoQC (Leger and Leonardi, 2019). For raw data processing, we made use of the snakemake pipeline provided by Oxford Nanopore (available at^1^). The pipeline performs read alignment, quantification, and differential expression analysis as well as abundance estimation in transcripts per million. For the detailed version of the environment used, see “Supplementary Material 1 versions.txt”. As a reference genome, we used the transcriptome cDNA reference provided by ensemble (Homo_sapiens.GRCh38.cdna.all.fa.gz, release 103). To quantify LT antigen expression, we added the sequence of the LT cDNA (GenBank: LC148302.1) to the assembly. As annotation, we used the corresponding GTF file where the LT antigen was added as an additional entry. For detailed sequence information, see Supplementary Figure 1 for the sequence.

### Differential Expression and Pathway Enrichment Analysis

Raw counts of the expressed transcripts were collected from the output of the snakemake pipeline. Further analysis was performed using the Bioconductor Package DESeq2 in R (Love et al., 2014); the code and data to perform the analysis were deposited at Zenodo under the DOI: 10.5281/zenodo.5031363. Because an exploratory approach was taken, we choose to set significance threshold to adjusted *p*-value < 0.1. *P*-values were adjusted using false discovery rate correction. Pairwise comparisons were performed between conditions (untreated and per flow cell and once across flow cell and cell lines). Gene set enrichment analysis was performed using the Bioconductor package fgsea (Sergushichev, 2016) with the C7 immune signature hallmarks, gene set version 7.2, by the Molecular Signatures Database (Subramanian et al., 2005; Liberzon et al., 2015).

### Mapping of Different Human Leukocyte Antigen Alleles

To quantify expression of the six known HLA alleles of each cell line, we implemented a two-pass quantification procedure, which quantifies transcriptome wide expression in the first pass and then uses prior knowledge to estimate allele specific HLA expression in the second pass. The data were then aggregated and analyzed. This allowed us to leverage the allele specific reference sequences (Robinson et al., 2019) and to mitigate the high error rates in nanopore data. In the first step, the known HLA alleles of each cell line were used separately to create a reference FASTA file, encompassing all described sequence variants of these alleles. We then mapped generated FASTQ files against this reference while also only counting primary alignments of a read. Afterward, read counting was performed for all reads with a sequence identity of 80% to the reference. This was done to achieve a conservative expression estimate and reduce false positive mappings. We further aggregated all reads mapping to the variants to a four-digit resolution, because higher genotyping resolutions are not known for the cell lines. In a second step, we gathered count data from previous mapping runs against the human genome reference and recalculated transcripts per million (TPM) with the counts generated in the first step. We thus arrive at a conservative estimate of the expression of HLA alleles in the investigated cell lines.

### Statistical Analysis

All graphs and statistical analysis for flow cytometry experiments were created by GraphPad Prism, version 8 (GraphPad Software, La Jolla, United States). The paired student’s *t*-test was used to compare intracellular and surface markers between cell lines treated with or without IFNγ as well as for the analysis of Western blot data. Data are reported as the median with standard error of the mean (SEM). Significance was determined as *p*-value ≤ 0.05.

## Results

### Interferon Gamma Increases Human Leukocyte Antigen Class I and PD-L1 in all Cell Lines, but Not Indoleamine-2,3-dioxygenase

**FIGURE 1:**
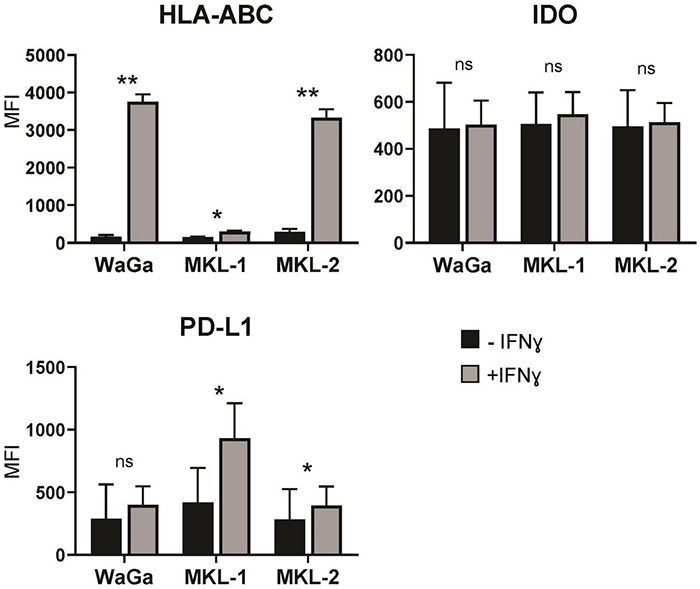
Merkel cell carcinoma cell lines respond to IFNγ by upregulating different proteins. After 72 h of treatment with or without IFNγ (3,000 U/ml), the Merkel cell carcinoma cell lines MKL-1, MKL-2, and WaGa were stained for human leukocyte antigen A, B, and C (HLA-ABC), indoleamine-2,3-dioxygenase (IDO), and programmed cell death 1 ligand 1 (PD-L1) and analyzed by flow cytometry. The average mean fluorescent intensity (MFI) of three to four independent experiments ± standard error is indicated. *p*-values were determined using the paired student’s *t*-test. *p < 0.05; **p < 0.01; ns, not significant.

To investigate the effect of IFNγ on important key players of the immune response, we measured the expression of (i) the HLA class I, whose loss can be a primary immune escape mechanism; (ii) PD-L1 as an important target for checkpoint inhibitors; and (iii) IDO as a key immunosuppressive enzyme. Antibody staining and FACS analysis of all three MCC cell lines were performed after 72-h incubation with IFNγ and as control without IFNγ. HLA class I was significantly upregulated in all three cell lines, whereas WaGa and MKL-2 cells displayed a stronger increase of HLA-ABC than MKL-1 cells (Figure 1). An increased expression of PD-L1 due to IFNγ was observed in all cell lines, but only for MKL-1 and MKL-2 with statistical significance. Staining for IDO showed no changes of the IDO status between the cell lines with and without IFNγ treatment. This indicates that all three cell lines are responsive to the cytokine but react differently to the treatment.

### Sequencing Runs

**FIGURE 2:**
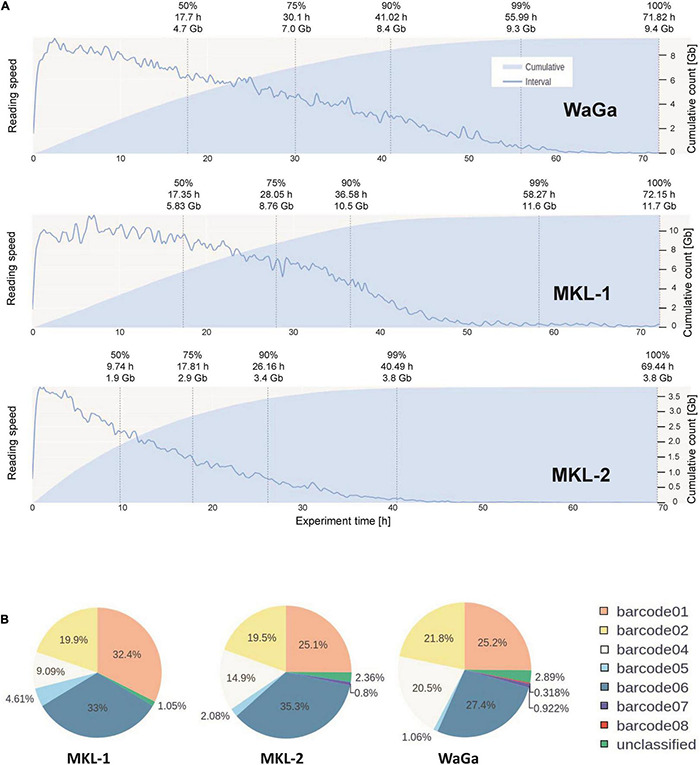
Functional performance of the Nanopore flowcells and barcodes. **(A)** Depiction of output over time of MKL-1, MKL-2, and WaGa cells. For each cell line, a new flow cell was loaded with the multiplexed library. The sequencing run lasted 72 h. The blue line indicates relative reading speed, i.e., bases per time, the light blue area represents cumulative reading output. **(B)** Barcode distribution of detected barcodes during the sequencing run of the three cell lines MKL-1, MKL-2, and WaGa. Barcode 3 is not displayed as it failed in the first run of MKL-1 and was skipped for further experiments. In the run of WaGa, we additionally tested barcode 8, which failed similar as barcode 3. The other barcodes (1, 2, 4, 5, 6, and 7) could be used according to their performance for multiplexing studies.

To comprehend the different transcriptional programs induced in the different cell lines by IFNγ, we sequenced RNA of the three MCC cell lines (MKL-1, MKL-2, and WaGa) *via* the Nanopore sequencing technology. Before RNA isolation, the cells were incubated with or without IFNγ for 24 h. During the sequencing runs, MKL-1 and WaGa produced comparable base pair (bp) outputs with 11.68 Gigabases (Gb) and 9.40 Gb after high-accuracy base calling. The experiments had similar pore counts available, at sequencing start with 1,446 for MKL-1 and 1,489 for WaGa. The flow cell dedicated for MKL-2 performed worse with only producing 3.29 Gb in total although similar pores were available for sequencing (1,480) and all experimental parameters equal but age of flow cell (Figure 2A). Overall, all three experiments generated high-quality Phred scores and high read lengths over their runtime, with MKL-1 producing a median read length of 1,217 bp and a median Phred(Q) of 11.98, and MKL-2 producing median read length of 630 bp and a median Q of 11.59. Finally, WaGa produced median read lengths of 683 bp and a median Q of 11.49. BC read yield showed biases in efficiency of each multiplexing BC. As for MKL-1, we observed a failure of BC 3 with no reads being generated from this BC. Of the remaining BCs, BC 6 performed best in total output. In further runs, we skipped BC 3 and assigned no sample to it. For MKL-2, BCs 1, 2, and 4, all assigned to the control group, showed good performance with BC 1, producing the best yield. In the treated group (BCs 5, 6, and 7), however, BC 6 again produced most reads with BC 5 and BC 7 almost failing. WaGa showed similar results, with the first three BCs (1, 2, and 4) performing equally well with BC 5 and BC 7, producing only 1% of reads. In addition, in this experiment, we had about 0.3% of erroneously assigned BC 8 detected. Unclassified counts were consistently low for all samples ranging from 0.8 to 2.0% of reads passing principal quality control (Figure 2B). Overall, Nanopore sequencing allowed for the quick and robust gathering of transcriptomics data from our samples. However, it is of note that some aspects like differentially BC efficacy and flow cell sensitivity are still a considerable factor.

### Differential Gene Expression

**FIGURE 3:**
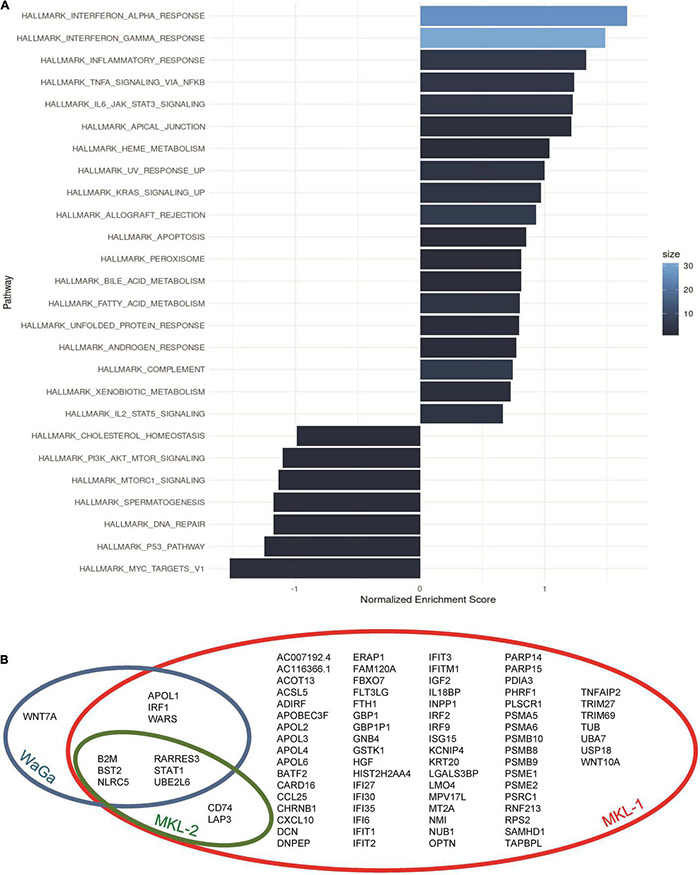
Pathway annotation and distribution of differentially expressed genes. **(A)** Hallmark pathway enrichment analysis of all used MCC cell lines (MKL-1, MKL-2, and WaGa): Data were pooled for the three cell lines in one plot. Gene set enrichment analysis was performed using the Bioconductor package fgsea with the immune signature hallmarks by Molecular Signatures Database. Size corresponds to gene count of how many expressed genes from the input overlap with the pathway. **(B)** The Venn diagram of the three cell lines gives an overview over shared differentially expressed genes between the cell lines MKL-1 (red circle), MKL-2 (green circle), and WaGa (blue circle).

Our primary goal was to detect differentially expressed genes in the MCC cell lines after treatment with IFNγ. Therefore, we performed a differential expression analysis of the Nanopore sequencing data using raw counts produced by our referenced pipeline. First, we pooled counts of all three cell lines, treating them as biological replicates, and corrected for batch effects. At an adjusted *p*-value < 0.1 and an absolute fold change >2, we could detect 61 genes that were differentially expressed (Supplementary Figure 1). To check for plausibility and to further investigate the role of the differentially expressed genes in terms of cancer biology, we performed a pathway enrichment analysis. Several pathways such as the inflammatory response pathway and the interleukine 6-janus kinase-signal transducer and activator of transcription (IL6-JAK-STAT3) pathway were enriched in the presence of IFNγ. Seven hallmark pathways were slightly downregulated, but most importantly, genes involved in IFNγ and IFNα pathways were induced (Figure 3A). Because these two pathways overlap to a large degree, this indicates that the data are sound and plausible.

**FIGURE 4:**
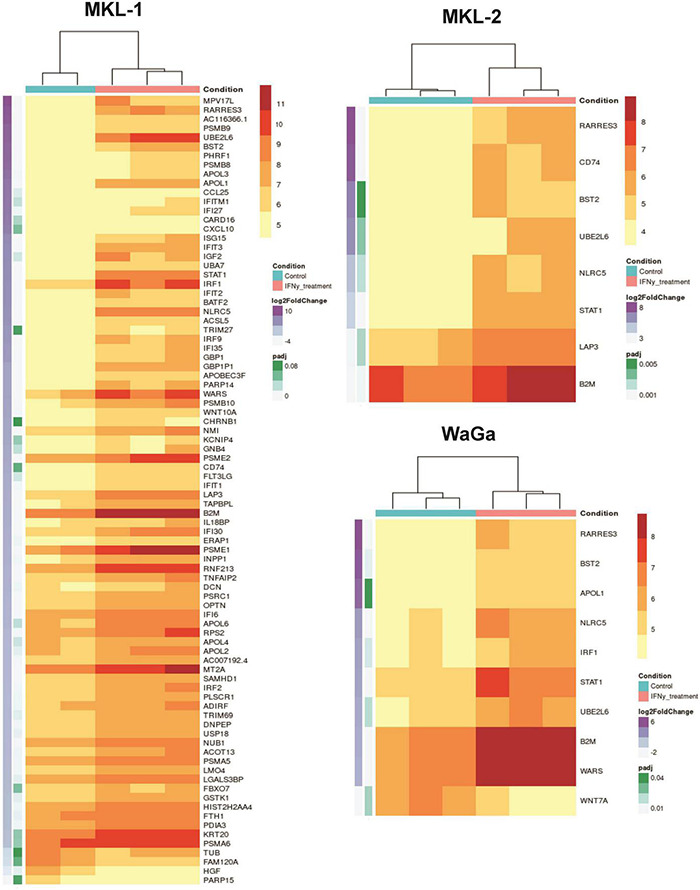
Differentially expressed genes of the cell lines MKL-1, MKL-2, and WaGa illustrated in heatmaps. Cell lines were incubated with IFNγ (blue top labels) or without IFNγ (pink top labels). Adjusted *p*-values are indicated in green and log_2_ fold change in purple on the left side. Listed genes were differentially expressed with a false discovery rate-adjusted *p*-value < 0.1 and an absolute fold change >2. A total of 86 genes was detected in MKL-1, eight genes in MKL-2, and 10 genes in WaGa cells. Heat scale represents vst-normalized counts using DESeq2.

To differentiate between the common IFNγ-induced program and a cell line–specific program, we investigated the cell lines individually and found 86 differentially expressed genes in cell line MKL-1, eight genes in MKL-2, and 10 genes in WaGa (Figure 2B). Six of these genes (UBE2L6, BST2, NLRC5, RARRES3, B2M, and STAT1) were differentially regulated in all three cell lines (Figure 3B). Furthermore, there was an overlap of two genes (CD74 and LAP3) between MKL-1 and MKL-2, whereas WaGA and MKL-1 showed differential expression for three genes (IRF1, WARS, and APOL1). Between WaGa and MKL-2, no individual overlap of differential gene expression could be detected. One gene (WNT7A) was differentially expressed uniquely in WaGa, whereas 74 of all detected genes were unique in MKL-1 and none for MKL-2. Obviously, the regulation (up- or downregulation) of each differentially expressed gene was of special interest. Thus, we performed a detailed examination of gene expression for each cell line in form of heatmaps (Figure 4) and volcano plots (Supplementary Figure 2). Most but not all differentially expressed genes were upregulated; WNT7A, HGF, PARP15, FAM120A, and TUB were significantly reduced in the presence of IFNγ.

The next step was to examine the role of the identified genes in the context of MCC and tumor biology. By manual annotation, we categorized the genes in the following groups according to their regulation, which we observed in the sequencing data: anti-tumoral genes, pro-tumoral genes, immune escape genes, and MCC-related genes (see Table 1).

**TABLE 1:** Overview of all differentially expressed genes categorized according to their activity in cancer biology.

Gene^1^	Protein name^2^	Anti-tumoral	Pro-tumoral	Immune escape	MCC related
AC007192.4 (↑)	Not annotated	–	–	–	–
AC116366.1	Not annotated	–	–	–	–
ACOT13 (↑)	Acyl–coenzyme A thioesterase 13	–	Liu et al., 2016	–	–
ACSL5 (↑)	Long-chain-fatty-acid–CoA ligase 5	Hung et al., 2017	–	–	–
ADIRF (↑)	Adipogenesis regulatory factor	–	–	–	–
APOBEC3F (↑)	DNA dC->dU-editing enzyme APOBEC-3F	Yen et al., 2017	–	–	–
APOL1 (↑)	Apolipoprotein L1	–	–	–	–
APOL2 (↑)	Apolipoprotein L2	–	–	–	–
APOL3 (↑)	Apolipoprotein L3	–	–	–	–
APOL4 (↑)	Apolipoprotein L4	–	–	–	–
APOL6 (↑)	Apolipoprotein L6	Yang et al., 2015	Liu et al., 2005	–	–
B2M (↑)	Beta-2-microglobulin	–	Maire et al., 2020	Maire et al., 2020	Zhang et al., 2021
BATF2 (↑)	Basic leucine zipper transcriptional factor ATF-like 2	Wen et al., 2014	–	–	–
BST2 (↑)	Bone marrow stromal antigen 2	–	Han et al., 2015	–	–
CARD16 (↑)	Caspase recruitment domain-containing protein 16	–	–	–	–
CCL25 (↑)	C-C motif chemokine 25	Liu et al., 2018	Chen et al., 2020; Niu et al., 2020	–	–
CD74 (↑)	HLA class II histocompatibility antigen gamma chain	Zhang et al., 2016	Greenwood et al., 2012; Zhang et al., 2014; Zeiner et al., 2018	Huang et al., 2013	Czech-Sioli et al., 2020
CHRNB1 (↑)	Acetylcholine receptor subunit beta	–	House et al., 2020	–	–
CXCL10 (↑)	C-X-C motif chemokine 10	Lo et al., 2010; Liu et al., 2011	Lo et al., 2010; Liu W. et al., 2020	–	Imaoka et al., 2019
DCN (↑)	Decorin	Järvinen and Prince, 2015	–	–	–
DNPEP (↑)	Aspartyl aminopeptidase	Geng et al., 2019	–	–	–
ERAP1 (↑)	Endoplasmic reticulum aminopeptidase 1	–	Stratikos, 2014; Steinbach et al., 2017	Steinbach et al., 2017	–
FAM120A (↓)	Constitutive coactivator of PPAR-gamma-like protein 1	Bartolomé et al., 2015	–	–	–
FBXO7 (↑)	F-box only protein 7	–	Lomonosov et al., 2011	–	–
FLT3LG (↑)	Fms-related tyrosine kinase 3 ligand	Abrahamsson et al., 2020; Pol et al., 2020	–	–	–
FTH1 (↑)	Ferritin heavy chain	Lobello et al., 2016; Aversa et al., 2017; Biamonte et al., 2018	Di Sanzo et al., 2011	–	–
GBP1 (↑)	Guanylate-binding protein 1	Lipnik et al., 2010; Britzen-Laurent et al., 2013	Mustafa et al., 2018; Ji et al., 2019; Wan et al., 2021	–	–
GBP1P1 (↑)	no protein, pseudogene	–	–	–	–
GNB4 (↑)	Guanine nucleotide-binding protein subunit beta-4	–	Wang B. et al., 2018; Gao et al., 2020a,b	–	–
GSTK1 (↑)	Glutathione S-transferase kappa 1	–	–	–	–
HGF (↓)	Hepatocyte growth factor	Owusu et al., 2017; Bonan et al., 2019	–	–	–
HIST2H2AA4 (↑)	Histone H2A type 2-A	–	–	–	–
IFI27 (↑)	Interferon alpha-inducible protein 27	–	Li et al., 2015; Wang H. et al., 2018; Chiang et al., 2019	–	–
IFI30 (↑)	Gamma-interferon-inducible lysosomal thiol reductase	–	Liu X. et al., 2020	Liu X. et al., 2020	–
IFI35 (↑)	Interferon-induced 35 kDa protein	–	–	–	–
IFI6 (↑)	Interferon alpha-inducible protein 6	–	Gupta et al., 2016; Liu Z. et al., 2020	–	–
IFIT1 (↑)	Interferon-induced protein with tetratricopeptide repeats 1	–	Pidugu et al., 2019	Pflügler et al., 2020	–
IFIT2 (↑)	Interferon-induced protein with tetratricopeptide repeats 2	Ohsugi et al., 2017	–	–	–
IFIT3 (↑)	Interferon induced protein with tetratricopeptide repeats 3	–	Niess et al., 2015	–	–
IFITM1 (↑)	Interferon-induced transmembrane protein 1	–	Yu et al., 2015; Yan et al., 2019		–
IGF2 (↑)	Insulin-like growth factor II	–	Kasprzak and Adamek, 2019	–	–
IL18BP (↑)	Interleukin-18-binding protein	–	Carbotti et al., 2013; Zhou et al., 2020	–	–
INPP1 (↑)	Inositol polyphosphate 1-phosphatase	–	Li et al., 2000; Li P. et al., 2019	–	–
IRF1 (↑)	Interferon regulatory factor 1	Bouker et al., 2005	–	Shao et al., 2019	–
IRF2 (↑)	Interferon regulatory factor 2	Kriegsman et al., 2019	–	–	–
IRF9 (↑)	Interferon regulatory factor 9	–	Luker et al., 2001; Brunn et al., 2021	–	–
ISG15 (↑)	Ubiquitin-like protein ISG15	D’Cunha et al., 1996; Zhou et al., 2017	Desai et al., 2006, 2012; Burks et al., 2014	–	–
KCNIP4 (↑)	Kv channel-interacting protein 4	–	–	–	Arora et al., 2020
KRT20 (↑)	Keratin, type I cytoskeletal 20	–	Wildi et al., 1999	–	–
LAP3 (↑)	Leucine aminopeptidase 3	–	Tian et al., 2014; Fang et al., 2019	–	–
LGALS3BP (↑)	Galectin-3-binding protein	–	Iacobelli et al., 1994; Rea et al., 1994	Läubli et al., 2014	Shao et al., 2013
LMO4 (↑)	LIM domain transcription factor LMO4	–	Sum et al., 2005; Wang W. et al., 2016	–	–
MPV17L (↑)	Mpv17-like protein	–	–	–	–
MT2A (↑)	Metallothionein-2	Pan et al., 2013	Jin et al., 2002; Kim et al., 2011	–	–
NLRC5 (↑)	NLR family CARD domain containing 5	Yoshihama et al., 2017, 2021	–	–	–
NMI (↑)	N-myc-interactor	Wang et al., 2017	Zhao et al., 2017	–	–
NUB1 (↑)	NEDD8 ultimate buster 1	Kito et al., 2001; Hosono et al., 2010; Zhang D. et al., 2020	–	–	–
OPTN (↑)	Optineurin	Liu et al., 2014	–	–	–
PARP14 (↑)	Protein mono-ADP-ribosyltransferase PARP14	–	Barbarulo et al., 2013; Yao et al., 2019	–	–
PARP15 (↓)	Protein mono-ADP-ribosyltransferase PARP15	–	–	–	–
PDIA3 (↑)	Protein disulfide-isomerase A3	–	Takata et al., 2016	Zhang H. et al., 2020	–
PHRF1 (↑)	PHD and RING finger domain-containing protein 1	Ettahar et al., 2013; Wang Y. et al., 2016	–	–	–
PLSCR1 (↑)	Phospholipid scramblase 1	–	Huang et al., 2020	–	–
PSMA5 (↑)	Proteasome subunit alpha type-5	–	Fu et al., 2019	–	–
PSMA6 (↑)	Proteasome subunit alpha type-6	–	Kakumu et al., 2017	–	–
PSMB10 (↑)	Proteasome subunit beta type-10	Rouette et al., 2016	–	–	–
PSMB8 (↑)	Proteasome subunit beta type-8	Kalaora et al., 2020	Kwon et al., 2016	–	Zhang et al., 2021
PSMB9 (↑)	Proteasome subunit beta type-9	Kalaora et al., 2020	Whitehead et al., 2005; Shoji et al., 2020	–	Zhang et al., 2021
PSME1 (↑)	Proteasome activator complex subunit 1	Wang et al., 2019	Sánchez-Martín et al., 2013	–	Kotowski et al., 2019
PSME2 (↑)	Proteasome activator complex subunit 2	–	Zheng et al., 2012	–	–
PSRC1 (↑)	Proline/serine-rich coiled-coil protein 1	–	–	–	–
RARRES3 (↑)	Retinoic acid receptor responder 3	Adelmann et al., 2016; Anderson et al., 2017	–	–	–
RNF213 (↑)	E3 ubiquitin-protein ligase RNF213	Wang et al., 2020	–	–	Yang et al., 2020
RPS2 (↑)	40S ribosomal protein S2	–	Chiao et al., 1992; Wang et al., 2009	–	–
SAMHD1 (↑)	Deoxynucleoside triphosphate triphosphohydrolase SAMHD1	Wang et al., 2014; Kodigepalli et al., 2017	Herold et al., 2017	–	–
STAT1 (↑)	Signal transducer and activator of transcription 1-alpha/beta	Meissl et al., 2017	Meissl et al., 2017; Zhang J. et al., 2020	Cerezo et al., 2018; Liu F. et al., 2020	–
TAPBPL (↑)	Tapasin-related protein	–	Lin et al., 2021	Hermann et al., 2015	–
TNFAIP2 (↑)	Tumor necrosis factor alpha-induced protein 2	–	Jia et al., 2016; Li et al., 2020	–	–
TRIM27 (↑)	Zinc finger protein RFP	–	Ma et al., 2016; Zhang et al., 2018; Xing et al., 2020	–	–
TRIM69 (↑)	E3 ubiquitin-protein ligase TRIM69	–	–	–	–
TUB (↓)	Tubby protein homolog	–	–	–	–
UBA7 (↑)	Ubiquitin-like modifier-activating enzyme 7	Feng Q. et al., 2008; Jiang et al., 2014; Lin et al., 2020	–	–	–
UBE2L6 (↑)	Ubiquitin conjugating enzyme E2 L6	–	Falvey et al., 2017	–	–
USP18 (↑)	Ubl carboxyl-terminal hydrolase 18	Hong et al., 2014	Tan et al., 2018; Diao et al., 2020	–	–
WARS (↑)	Tryptophanyl-tRNA synthetase	–	Adam et al., 2018	Adam et al., 2018	–
WNT10A (↑)	Protein Wnt-10a	–	Long et al., 2015; Li J. et al., 2019	–	Harms et al., 2013
WNT7A (↓)	Wnt family member 7A	Yoshioka et al., 2012; Wu et al., 2018	–	–	–

*^1^Genes with differential gene expression in form of upregulaton are marked with ↑, and downregulation is marked with ↓.*

*^2^Recommended protein name by UniProtKB (www.uniprot.org).*

According to this classification, we detected 35 genes associated with anti-tumoral effects (e.g., resulting in an increase in the numbers of infiltrating lymphocytes or inhibition of tumor cell growth). In contrast, we found 49 genes associated with pro-tumoral effects (e.g., genes supporting tumor growth or increasing survival and migration of cancer cells). A lot of these pro-tumoral genes overlapped with the group of potential tumor immune escape genes, of which 11 genes showed significantly increased gene expression under IFNγ treatment: CD74, STAT1, B2M, IRF1, WARS, IFI30, TAPBPL, IFIT1, PDIA3, ERAP1, and LGALS3BP. Of note, in some cases, anti-tumoral genes were downregulated resulting in a pro-tumoral effect or, on the contrary, the expression of pro-tumoral genes was decreased leading to anti-tumoral activity. In addition, we found 14 genes, which could have both anti- and pro-tumoral activity. Which kind of effect these genes actually had was often cell-type or cancer-type dependent. With respect to the MCC-related genes, we identified 10 genes that were already known to play a role in MCC, such as genes involved in the integration site of the MCPyV or acting as predictive biomarker. Of these genes, B2M was the only one that was differentially expressed in all three cell lines while CD74 expression was altered in MKL-1 and MKL-2, whereas all other genes related to MCC were regulated by IFNγ to MKL-1 alone.

### Human Leukocyte Antigen Expression Is Increased on mRNA Level by Interferon Gamma

**FIGURE 5:**
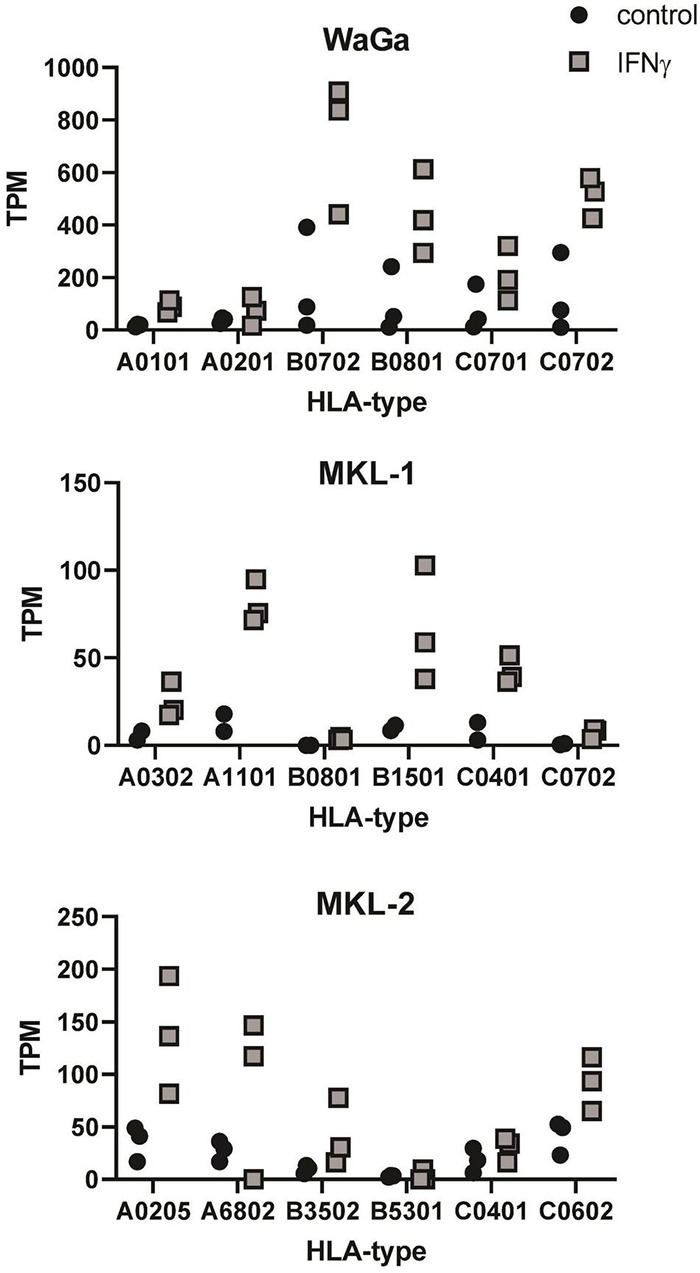
Detection of HLA expression after IFNγ incubation on mRNA level. Transcripts per million (TPM) values of six different HLA alleles after IFNγ treatment (gray squares) or without IFNγ incubation (black dots) are depicted for each MCC cell line. TPM values were acquired from our sequencing output using a new two-pass quantification method. For each cell line, three independent experiments were performed (two for MKL-1 control), so that each symbol represents one replicate of the respective cell line.

The relatively high error rate of Nanopore sequencing results in poor mapping of the individual reads in the HLA regions of the reference genomes. Hence, we implemented a two-pass quantification procedure to exploit the up-front knowledge about the HLA-haplotypes of the used cell lines. With this two-pass quantification step, we were able to verify the upregulation of HLA-specific expression following an IFNγ treatment in the three MCC cell lines (Figure 5). The calculated TPM values of the six different HLA alleles were considerably higher for the IFNγ-treated group across all three cell lines, despite slight differences in single replicates or subtypes. However, because the different approaches on how read counts were generated, the data were not included in the statistical analysis of the whole transcriptomes described above. Instead, we decided to provide a separate estimate of the allele expression to make clear that these are two different avenues of analysis.

The relatively high error rate of Nanopore sequencing reads results in ubiquitous mapping of reads between loci that share a high degree of sequence similarity. This especially presents an issue with the HLA loci, because there is considerable sequence homology between the alleles and supertypes (HLA-A, B, and C). We found that, by using standard alignment methods, it is hard to allocate reads to the correct allele and thus decided to implement our two-pass quantification procedure, making use of the prior knowledge of the alleles present in our cell lines of interest.

### Validation of Differential Gene Expression on Protein Level

**FIGURE 6:**
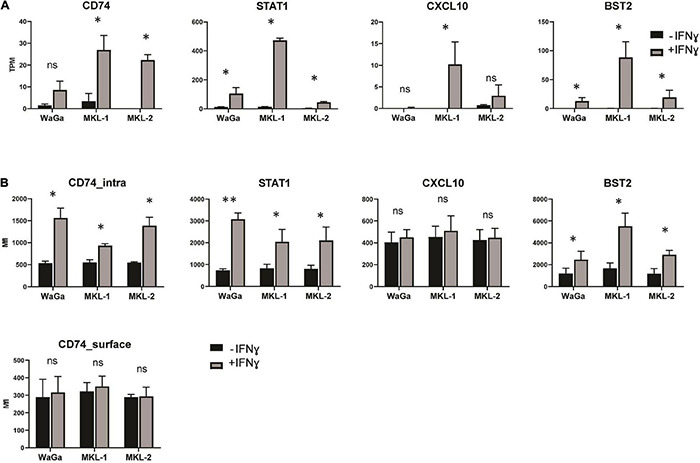
Validation of differential gene expression in MCC cell lines on protein level and mRNA level. **(A)** TPM values were acquired from our sequencing output. The data represent mean values ± SEM from three independent experiments (two for MKL-1 control). **(B)** After 72 h of treatment with or without IFNγ (3,000 U/ml), the Merkel cell carcinoma cell lines MKL-1, MKL-2, and WaGa were stained for signal transducer and activator of transcription 1-alpha/beta (STAT1), bone marrow stromal antigen 2 (BST2), C-X-C motif chemokine 10 (CXCL10), and HLA class II histocompatibility antigen gamma chain (CD74) and analyzed by flow cytometry. The average MFI of three independent experiments ± standard error is indicated. *p*-values were determined using the paired student’s *t*-test. *p < 0.05; ^**^p < 0.01; ns, not significant.

To confirm our transcriptomic findings (Figure 6A) on protein level, we exemplarily studied four differentially expressed genes (STAT1, BST2, CXCL10, and CD74) using flow cytometry (Figure 6B). Therefore, the three MCC cell lines were incubated with IFNγ (3,000 U/ml) for 72 h, and, afterward, antibody staining of the selected genes was performed. We chose genes that were differentially expressed on mRNA level in all three cell lines (STAT1 and BST2) and genes that were upregulated in only one (CXCL10 in MKL-1) or two (CD74 in MKL-1 and MKL-2) of the MCC cell lines. Our findings in flow cytometry did mainly match with our transcriptomic data: We observed significantly increased STAT1 and BST2 level in all three cell lines. The molecule CXCL10 showed no significant differences between ± IFNγ in all tested cell lines. CD74 was stained as surface molecule but did not show any differences between ± IFNγ on protein level. Nevertheless, intracellular staining of MCC cells incubated with IFNγ led to an increase of CD74 in all three cell lines. These data indicate that the differences on mRNA-expression level translate to correspondingly different amounts of protein for most, but not all gene products.

### Large T Antigen Expression Under Interferon Gamma Treatment

**FIGURE 7:**
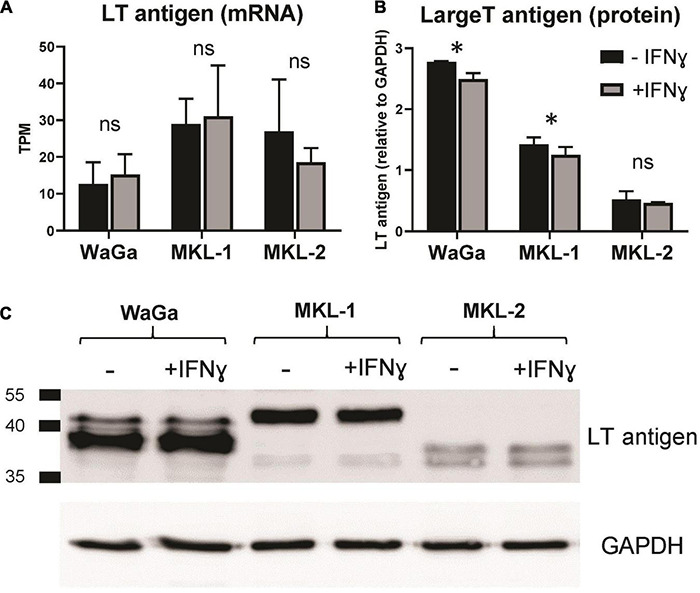
Large T antigen expression before and after IFNγ treatment. **(A)** TPM values were acquired from our sequencing output. The data represent mean values ± SEM from three independent experiments (two for MKL-1 control). **(B)** LT antigen expression was analyzed on protein level *via* Western blot analysis. Expression levels are depicted relative to the loading control GAPDH. The average of three independent experiments ± standard error is indicated. *p*-values were determined using the paired student’s *t*-test. *p < 0.05; ^**^p < 0.01; ns, not significant. **(C)** Representative exposure of one western blot. Protein size standard is indicated in kilodalton.

Expression of the LT antigen is a crucial factor for MCC development. LT antigen was shown to be regulated by IFNγ on protein level (Willmes et al., 2012); hence, we tested whether this regulation also takes place on mRNA level. TPM values were acquired from our sequencing output. The expression of the LT antigen was not substantially altered by IFNγ on mRNA level (Figure 7A). To study this result on protein level, we performed a Western Blot analysis with cell lysates treated with or without IFNγ for 72 h (Figures 7B,C). We detected a small but significant decrease of LT antigen after IFNγ incubation in the cell lines WaGa and MKL-1, whereas LT antigen expression was not significantly altered in MKL-2.

## Discussion

In this study, single-molecule Nanopore sequencing has been used to analyze the transcriptome of MCC cells. Previously, whole transcriptome and genome studies of MCC using the second-generation sequencing could demonstrate distinct mutation spectra and expression profiles between virus-positive and virus-negative MCC (Starrett et al., 2017). Czech-Sioli et al. used genome sequencing *via* the Nanopore platform to perform high-resolution analysis of the genomic structure of the integration site of the MCPyV into the host genome of MCC cell lines and primary tumors. In addition, they employed the technology to reveal copy numbers and integration pattern of the MCPyV (Czech-Sioli et al., 2020), underlining the increasing popularity of Nanopore sequencing in cancer research. However, no studies on the transcriptome-wide response to key cytokines had been conducted so far.

### Immune Escape Genes and Human Leukocyte Antigen Class I Upregulation

As shown for IFNγ in various other settings, our sequencing studies found numerous differentially expressed genes that have been associated with both anti-tumoral and pro-tumoral effects. These data indicate the different modes of action of IFNγ: On the one hand, it acts highly tumor suppressive; on the other hand, it dampens the immune response by synthesis of inhibitory molecules (John and Darcy, 2016). Furthermore, we detected 11 immune escape genes that were significantly increased by IFNγ, suggesting that these are involved in the formation of an immunosuppressive tumor microenvironment counteracting the inflammatory effect of IFNγ (Mojic et al., 2017). Targeting these genes could improve the responsiveness to checkpoint inhibitor therapy and increase immunosurveillance and survival of MCC patients.

Downregulation of HLA class I and upregulation of immune checkpoint proteins (PD-1, CTLA-4, and PD-L1) are known to help the tumor to escape the control of the immune system. Paulson et al. reported reduced expression of HLA class I in MCC cell lines, which was reversible by IFNγ treatment (Paulson et al., 2014). In our flow cytometry experiments, we could also detect a clear upregulation of HLA class I surface expression in the presence of IFNγ in MKL-2 and WaGa cells, whereas MKL-1 cells showed only a slight increase of HLA class I. This is noteworthy because MKL-1 had the highest number of differentially expressed genes after IFNγ treatment. Expression levels of HLA have previously been shown to be low in MKL-1 cells, intermediate in WaGa cells, and high in MKL-2 cells (Ritter et al., 2017). In our flow cytometry experiments, without the addition of IFNγ, we could detect only low levels of HLA in WaGa and MKL-1 cells and higher levels in MKL-2 cells. Increased HLA induced by IFNγ, which counteracts HLA downregulation by the tumor, could contribute to higher effectiveness of immunotherapies in MCC patients. In awareness of the high error rates of Nanopore sequencing and the variability of the HLA allele–specific expression, we were able to verify the changes in HLA expression behavior, previously detected by flow cytometry, in the sequencing data. This strengthens the usability of Nanopore sequencing for highly variable alleles and the measurement of transcriptome-wide response to key cytokines. However, without prior HLA-typing of the subject, we consider that the third-generation sequencing still has difficulties to identify and quantify expression of highly polymorphic HLA loci.

### Immune Checkpoints Are Influenced by Interferon Gamma Treatment

We studied the effect of IFNγ on the immune checkpoint PD-L1 and the key immune suppressive molecule IDO. IDO is of special interest for MCC as it has been shown that IDO expression is associated with MCPyV status and prognosis (Wardhani et al., 2019). To date, the effect of IFNγ on IDO has not been studied in MCC. We could not detect any alteration of IDO in all cell lines neither on mRNA nor on protein level. In contrast, IDO has been reported to be upregulated by IFNγ in the tumor microenvironment of melanoma (Spranger et al., 2013) and several other cell types, such as dendritic cells (Jürgens et al., 2009). Interestingly, our sequencing data show that WARS, which can protect cancer cells from IDO-mediated tryptophan depletion (Adam et al., 2018), is highly upregulated on mRNA level in the presence of IFNγ in all cell lines, underlining the pro-inflammatory activity of IFNγ in this context.

Interferon gamma is known to contribute to tumor immune resistance by inducing the expression of immune checkpoint receptors and their ligands (Benci et al., 2016). We could demonstrate a slight upregulation of PD-L1 in MKL-1 and MKL-2 cells by flow cytometry analysis. Another interesting protein associated with inhibitory effects on T cells is Tapasin-related protein, encoded by the gene TAPBPL. TAPBPL was highly upregulated in IFNγ-exposed MKL-1 cells. Tapasin-related protein is a T cell co-inhibitory molecule identified in 2021 (Lin et al., 2021), which is expressed on cancer cells and antigen-presenting cells as well as the corresponding receptor on activated CD4 and CD8 T cells. As a new immune checkpoint molecule, it might be an interesting target for cancer therapy.

### Alteration in Large T Antigen Expression on Protein but Not on mRNA Level

Large T antigen mRNA expression was not significantly altered by IFNγ treatment in all three MCC cell lines. However, our studies of the LT antigen on protein level showed small but significant decreased expression in WaGa and MKL-1, whereas no alteration could be detected in MKL-2. We hypothesize that IFNγ might have a destabilization effect only on protein level in WaGa and MKL-1 cells, which is not detectable in the mRNA profile. Interestingly, Willmes et al. described a clear reduction of the LT antigen on protein level in WaGa cells, whereas MKL-1 and MKL-2 cells showed no change in LT protein expression, when analyzed after 7 days of IFNγ treatment (Willmes et al., 2012). This discrepancy may be attributed to the kinetics of LT antigen regulation, which may yield different results at different time points.

### Oxford Nanopore Sequencing for Transcriptomics

In this study, we strived to elucidate the biological response of MCC cell lines to IFNγ stimulation while also trying to ascertain whether the expression studies of large eukaryotic transcriptomes are sensible and feasible using the Nanopore platform. We especially were interested to produce reliable and reproducible data at the current stage of the yet relatively new Nanopore system. There have been several benchmarking studies on Nanopore sequencing in the context of cDNA or direct RNA sequencing that discuss the system in a benchmark-type situation (Workman et al., 2019; Chen et al., 2021). Our goal, however, was to test practicability, robustness, and reproducibility in an applied setting. We found that the Nanopore system offers great potential for researchers to get access to in-house–produced sequencing data with relatively low entry costs and virtually no maintenance costs. These advantages come, however, at the price of high hardware requirements, a necessity of sufficient bioinformatics infrastructure and a fast-evolving system, in which laboratory protocols, software versions, and specifications as well as the recommendations of the manufacturer change with higher frequency as compared to firmly established sequencing environments as Illumina. We especially had issues pinpointing specific points of failure in experimental procedures that were virtually identical but for the flow cell used. Documentation on troubleshooting was sparse and hard to find at the time of our experiments. It is, however, noteworthy that, since then, Oxford Nanopore has greatly improved its available training resources.

Pore count at the beginning of an experiment appeared to be a good indicator of the possible total sequencing output for us; however, this was not true for flow cells that had been stored for longer periods and were approaching expiration date. In the experiment with MKL-2, we observed a rapid drop off of sequencing output after 24 h (Figure 2A) although the flow cell was older but still above warranty level and had ample pores available. In addition, BC performance widely varied. The good performance of BC 1 in terms of yield could be replicated across all three experiments, similar to BC 6. BC 3 failed completely—an issue the broader community was aware of, and we became aware of during troubleshooting. However, BC 5 and BC 7 bordered on failure as well over all three flow cells.

The MinION Sequencing platform by Oxford Nanopore is a rapidly evolving technology. Troubleshooting was challenging for us at times. The biological nature of embedded pores creates a necessity for stringent experiment planning, because extended storage times appeared to have a negative effect on yield. However, the access to sequencing data within 3 days from library prep to finished data is very attractive. In addition, long reads allow the accurate quantification of gene expression even at low sequencing depth because even in an cDNA assay, each reverse-transcribed molecule came from a native poly(A)-mRNA molecule with no fragmentation bias. Overall, the system is under active development and Q 20 chemistry (99% base call accuracy) has already been announced while constant improvements are made to hardware and protocols. The system offers a great entry point to sequencing, even for smaller laboratories, and allows rapid quantification of the expression landscape of a sample.

### Validation of Transcriptomic Data on Protein Level

To validate our Nanopore sequencing data, we further analyzed the expression of STAT1, CXCL10, BST2, CD74, and the LT antigen on protein level. We detected a significant increase of STAT1 and BST2 in all three cell lines, as we had observed on mRNA level. STAT1 is itself involved in the IFNγ signaling pathway and both STAT1 and BST2 are known to be IFN inducible (Soler et al., 2003; Blasius et al., 2006; Krause et al., 2006; Yoo et al., 2011). In addition, CD74 significantly increased on protein level for all three cell lines. In our sequencing findings, increased CD74 was only significant for MKL-1 and MKL-2, whereas the observed upregulation in WaGa did not meet significance. Interestingly, CD74 showed significant regulation only when stained intracellularly; surface expression of CD74 on the MCC cell lines was, in general, lower and completely unaltered by IFNγ. The transcript of the CD74 gene forms invariant chain of the immature HLA class II complex. The cell surface form, in contrast, acts as receptor for macrophage migration inhibitory factor (Imaoka et al., 2019) and is involved in tumorigenesis (Liu et al., 2016). This complex post-translational processing is probably the reason for the discrepancy between mRNA and intracellular expression, on the one hand, and surface expression, on the other hand.

C-X-C motif chemokine 10 was strongly increased on mRNA level in one MCC cell line incubated with IFNγ (MKL-1); nevertheless, we could not detect any significant differences on protein level, and the signal was relatively low. Because CXCL10 is a chemokine, we speculate that it is rapidly secreted and does not accumulate to detectable levels intracellularly, especially because no secretion-blocking substances like brefeldin A or monensin were added.

In summary, we could demonstrate the complexity of IFNγ responses mirrored by numerous differentially expressed genes induced by IFNγ, mainly with anti- or pro-tumoral effects or even both. In this context, it would be interesting to sequence primary MCC cell cultures and to examine their response to IFNγ because this could better represent *in vivo* conditions. The identified immune escape genes are of special interest for further studies as these could deliver valuable information about how the tumor circumvents the control of the immune system. Targeting the genes involved in immune evasion and tumor progression could be beneficial for the clinical outcome of MCC patients.

## Data Availability Statement

The datasets presented in this study can be found in online repositories. The names of the repository/repositories and accession number(s) can be found below: https://zenodo.org/record/5031363#.YVQtx31CSUk, DOI: 10.5281/zenodo.5031363; https://zenodo.org/record/5645039#.YYPJNrso9H4, DOI: 10.5281/zenodo.5645039.

## Author Contributions

JD: conceptualization. TS and JD: experimental procedures. CL, AW, and JV: data processing and analysis. JD and JV: funding acquisition and supervision. TS and CL: investigation and writing—original draft. CL, TS, AW, CB, JD, and JV: writing—review and editing. All authors have read and agreed to the published version of the manuscript.

## Conflict of Interest

The authors declare that the research was conducted in the absence of any commercial or financial relationships that could be construed as a potential conflict of interest.

## Publisher’s Note

All claims expressed in this article are solely those of the authors and do not necessarily represent those of their affiliated organizations, or those of the publisher, the editors and the reviewers. Any product that may be evaluated in this article, or claim that may be made by its manufacturer, is not guaranteed or endorsed by the publisher.

## Footnotes

1
https://github.com/nanoporetech/pipeline-transcriptome-de/commit/47c850bd320031ed1e2b36cd631f287e0d98e464
