# Supplementary material for: Single-Molecule RNA Sequencing Reveals IFNγ-Induced Differential Expression of Immune Escape Genes in Merkel Cell Polyomavirus–Positive MCC Cell Lines
Source: Front Microbiol. 2021 Dec 22;12:785662. doi: 10.3389/fmicb.2021.785662 (PMC8727593; doi:10.3389/fmicb.2021.785662)
Supplement: Supplementary file 1 [file Data_Sheet_1.PDF]

>LargeT-Antigen

ATGGATTTAGTCCTAAATAGGAAAGAAAGAGAGGCTCTCTGCAAGCTTTTAGAGATTGCTCCTAATTGTTATGGCAACATC
CCTCTGATGAAAGCTGCTTTCAAAAGAAGCTGCTTAAAGCATCACCCTGATAAAGGGGGAAATCCTGTTATAATGATGGAA
TTGAACACCCTTTGGAGCAAATTCCAGCAAAATATCCACAAGCTCAGAAGTGACTTCTCTATGTTTGATGAGGTTGACGAG
GCCCCTATATATGGGACCACTAAATTCAAAGAATGGTGGAGATCAGGAGGATTCAGCTTCGGGAAGGCATACGAATATGG
GCCCAATCCATACGGGACCAACTCAAGATCCAGAAAGCCTTCCTCCAATGCATCCAGGGGAGCCCCCAGTGGAAGCTCAC
CACCCCACAGCCAGAGCTCTTCCTCTGGGTATGGGTCCTTCTCAGCGTCCCAGGCTTCAGACTCCCAGTCCAGAGGACCCG
ATATACCTCCCGAACACCATGAGGAACCCACCTCATCCTCTGGATCCAGTAGCAGAGAGGAGACCACCAATTCAGGAAGA
GAATCCAGCACACCAAATGGAACCAGTGTACCTAGAAATTCTTCCAGAACGGATGGCACCTGGGAGGATCTCTTCTGCGAT
GAATCACTTTCCTCCCCTGAGCCTCCCTCGTCCTCTGAGGAGCCTGAGGAGCCCCCCTCCTCAAGAAGCTCGCCCCGGCAG
CCCCCGTCTTCCTCTGCCGAGGAGGCCTCGTCATCTCAGTTTACAGATGAGGAATGCAGATCCTCCTCCTTCACCACCCCGA
AGACCCCTCCTCCATTCTCAAGAAAGCGAAAATTTGGGGGGTCCCGAAGCTCTGCAAGCTCTGCTAGTTCAGCAAGTTTTA
CAAGCACTCCACCAAAGCCAAAAAAGAACAGAGAAACTCCTGTTCCTACTGATTTTCCTATTGATCTTTCTGATTATCTTAGC
CATGCTGTATATAGTAATAAAACAGTAAGTTGTTTTGCCATTTATACTACTTCTGATAAAGCTATAGAGTTATATGATAAGA
TTGAGAAATTTAAAGTTGATTTTAAAAGCAGGCATGCCTGTGAATTAGGATGTATTTTATTGTTTATAACTTTATCAAAGCA
TAGAGTATCTGCTATTAAGAATTTTTGCTCTACCTTCTGCACTATAAGCTTTTTAATCTGTAAAGGAGTGAATAAGATGCCT
GAAATGTATAATAATTTATGTAAGCCCCCTTACAAATTACTGCAAGAGAATAAGCCACTGCTCAATTATGAATTTCAAGAAA
AAGAAAAAGAGGCCAGCTGTAATTGGAATTTAGTTGCTGAATTTGCTTGTGAATATGAGCTAGACGACCACTTTATTATCT
TAGCCCATTATCTAGACTTTGCAAAACCATTTCCTTGCCAAAAGTGTGAAAACAGATCTCGCCTCAAACCTCACAAGGCTCA
TGAGGCTCATCATTCTAATGCTAAGCTATTTTATGAATCTAAATCTCAGAAAACCATTTGCCAACAAGCCGCAGACACTGTT
CTAGCCAAAAGGAGGTTAGAGATGCTGGAAATGACCAGAACAGAAATGCTATGTAAGAAGTTTAAGAAGCACCTAGAGA
GATTAAGAGATTTAGATACAATAGATTTATTGTATTATATGGGTGGTGTGGCTTGGTATTGTTGTTTATTTGAAGAGTTTGA
AAAGAAGCTGCAGAAAATTATTCAATTATTAACAGAGAATATACCTAAGTATAGAAACATTTGGTTTAAAGGGCCTATTAA
CAGTGGAAAAACAAGCTTTGCTGCAGCCTTAATAGATTTGCTAGAAGGGAAGGCCTTGAATATAAACTGTCCATCAGATAA
ACTACCTTTTGAACTAGGATGTGCTTTGGATAAATTTATGGTTGTTTTTGAGGATGTGAAAGGGCAAAATAGCCTAAATAA
AGATCTGCAACCAGGGCAAGGAATAAATAACCTTGATAACTTAAGAGATCATCTAGATGGTGCTGTAGCTGTAAGCTTAG
AGAAGAAGCATGTGAATAAAAAGCATCAGATTTTTCCTCCTTGTATTGTTACTGCTAATGATTACTTTATTCCCAAAACATTA
ATAGCAAGATTTAGTTATACTTTACACTTTTCCCCAAAGGCAAATCTAAGAGATTCCCTGGATCAGAACATGGAAATAAGA
AAAAGAAGAATTCTTCAAAGTGGAACCACTTTATTGCTTTGTCTTATTTGGTGCTTGCCTGATACAACCTTTAAGCCTTGCTT
ACAAGAAGAAATTAAAAACTGGAAGCAAATTTTACAGAGTGAGATATCATATGGTAAATTTTGTCAAATGATAGAAAATGT
AGAAGCTGGTCAGGACCCTCTGCTCAATATTCTTATTGAGGAAGAGGGCCCTGAGGAAACTGAAGAAACCCAAGATTCTG
GTACTTTTTCTCAATAA

Supplementary figure S1: Fasta formated cDNA sequence of the used Large T Antigen sequence. The genoic sequence was taken from Genbank: LC148302.1, The intron was removed to generate the Large T Antigen mRNA

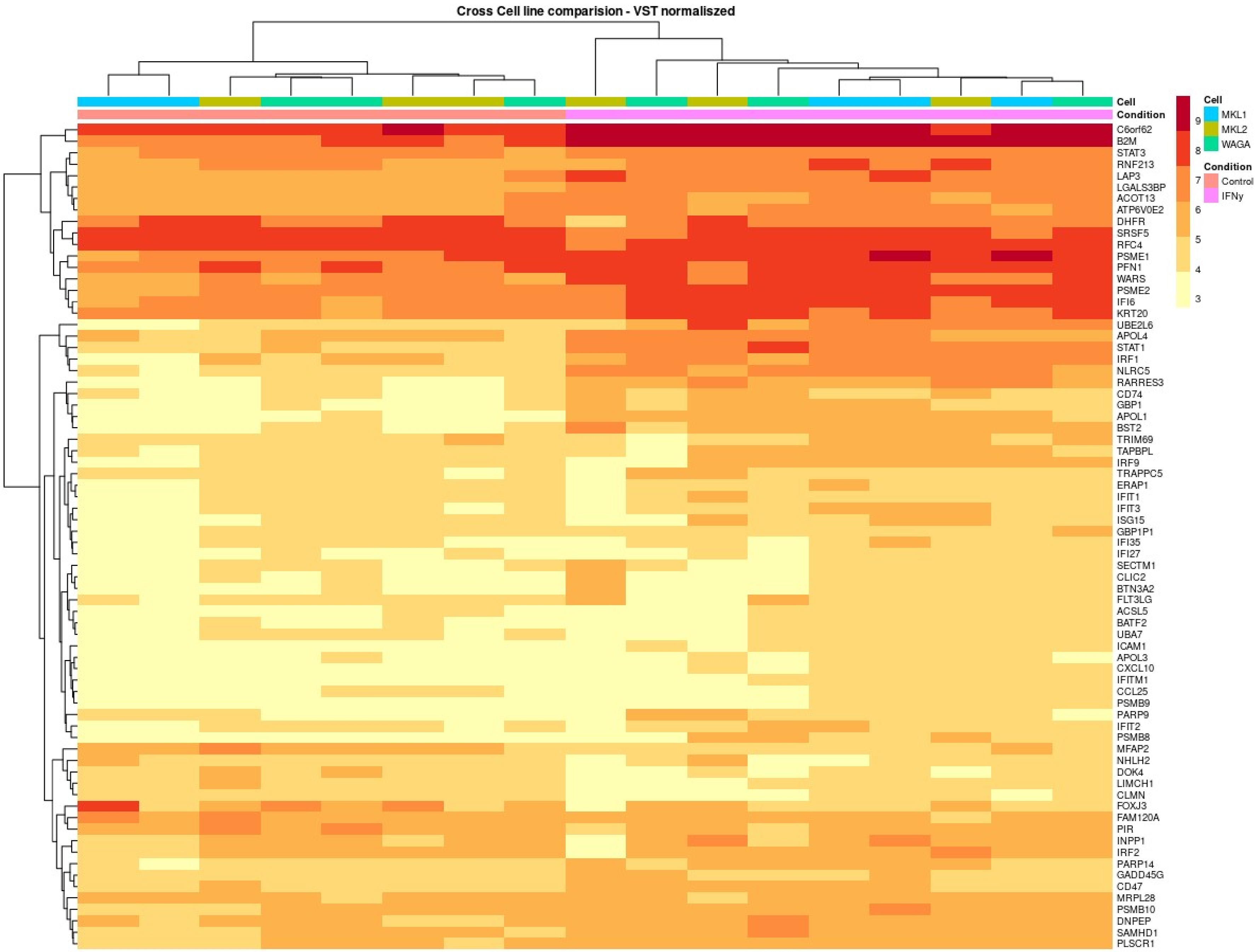

Supplementary figure S2: **Heatmap of global differential expression analysis of the three cell lines MKL-1, MKL-2 and WaGa.** Counts of all three cell lines were pooled, treated as biological replicates, and blocked against effects caused by cell line differences. The control group without IFNɣ is marked in red, the IFNɣ-treated group in purple. At an adjusted p-value < 0.1 and an absolute fold change >2 we found that 61 genes were differentially expressed. Heat scale represents vst-normalized counts using DESeq2.

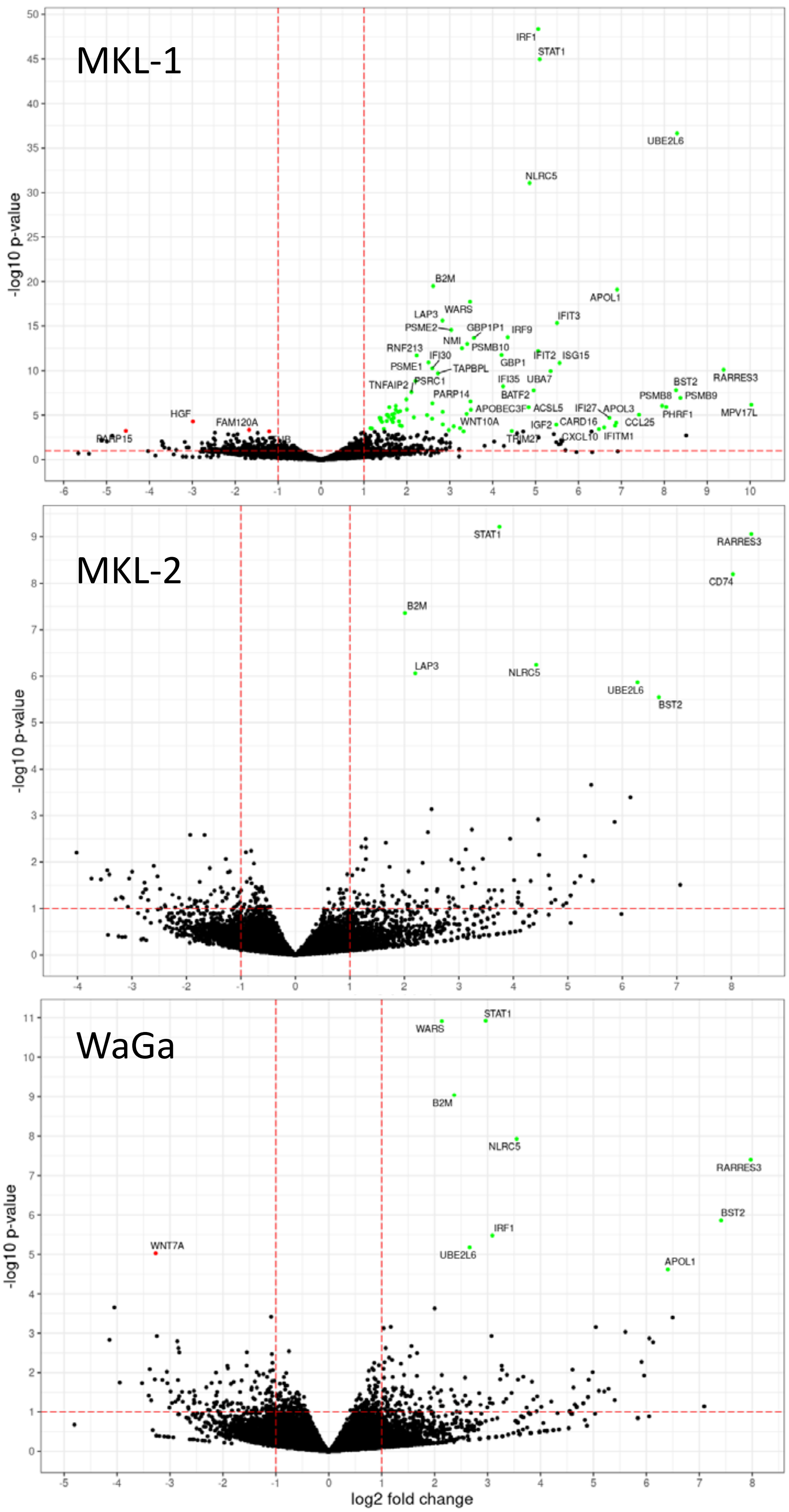

Supplementary figure S3: Volcano plots of MKL-1, MKL-2 and WaGa: The Y-axis represents the unadjusted p-value. Genes which are differentially expressed (according to the adjusted p-value) are represented by green (upregulated) or red dots (downregulated).
