# Supplementary material for: Single-Molecule RNA Sequencing Reveals IFNγ-Induced Differential Expression of Immune Escape Genes in Merkel Cell Polyomavirus–Positive MCC Cell Lines
Source: Front Microbiol. 2021 Dec 22;12:785662. doi: 10.3389/fmicb.2021.785662 (PMC8727593; doi:10.3389/fmicb.2021.785662)
Supplement: Supplementary file 2 [file Data_Sheet_2.pdf]

# Name	Version	Build	Channel
_libgcc_mutex	0.1	conda_forge	conda-forge
_openmp_mutex	4.5	1_gnu	conda-forge
_r-mutex	1.0.1	anacondar_1	conda-forge
binutils_impl_linux-64	2.35.1	h193b22a_2	conda-forge
binutils_linux-64	2.35	h67ddf6f_30	conda-forge
bioconductor-annotate	1.68.0	r40hdfd78af_1	bioconda
bioconductor-annotationdbi	1.52.0	r40hdfd78af_1	bioconda
bioconductor-biobase	2.50.0	r40hd029910_1	bioconda
bioconductor-biocfilecache	1.14.0	r40hdfd78af_1	bioconda
bioconductor-biocgenerics	0.36.0	r40hdfd78af_1	bioconda
bioconductor-biocparallel	1.24.1	r40h399db7b_0	bioconda
bioconductor-biomart	2.46.3	r40hdfd78af_0	bioconda
bioconductor-biostrings	2.58.0	r40hd029910_1	bioconda
bioconductor-delayedarray	0.16.3	r40hd029910_0	bioconda
bioconductor-deseq2	1.30.1	r40h399db7b_0	bioconda
bioconductor-dexseq	1.36.0	r40hdfd78af_1	bioconda
bioconductor-drimseq	1.18.0	r40hdfd78af_1	bioconda
bioconductor-edger	3.32.1	r40h399db7b_0	bioconda
bioconductor-genefilter	1.72.1	r40hba52eb8_0	bioconda
bioconductor-geneplotter	1.68.0	r40hdfd78af_1	bioconda
bioconductor-genomeinfodb	1.26.4	r40hdfd78af_0	bioconda
bioconductor-genomeinfodbdata	1.2.4	r40hdfd78af_2	bioconda
bioconductor-genomicalignments	1.26.0	r40hd029910_1	bioconda
bioconductor-genomicfeatures	1.42.2	r40hdfd78af_0	bioconda
bioconductor-genomicranges	1.42.0	r40hd029910_1	bioconda
bioconductor-iranges	2.24.1	r40hd029910_0	bioconda
bioconductor-limma	3.46.0	r40hd029910_1	bioconda
bioconductor-matrixgenerics	1.2.1	r40hdfd78af_0	bioconda
bioconductor-rhtslib	1.22.0	r40hd029910_1	bioconda
bioconductor-rsamtools	2.6.0	r40h399db7b_1	bioconda
bioconductor-rtracklayer	1.50.0	r40h7f5ccec_2	bioconda
bioconductor-s4vectors	0.28.1	r40hd029910_0	bioconda
bioconductor-stager	1.12.0	r40hdfd78af_1	bioconda
bioconductor-summarizedexperiment	1.20.0	r40hdfd78af_1	bioconda
bioconductor-xvector	0.30.0	r40hd029910_1	bioconda
bioconductor-zlibbioc	1.36.0	r40hd029910_1	bioconda
boost-cpp	1.74.0	hc6e9bd1_2	conda-forge
bwidget	1.9.14	ha770c72_0	conda-forge
bzip2	1.0.8	h7f98852_4	conda-forge
c-ares	1.17.1	h7f98852_1	conda-forge
ca-certificates	2020.12.5	ha878542_0	conda-forge
cairo	1.16.0	h6cf1ce9_1008	conda-forge
certifi	2020.12.5	py39hf3d152e_1	conda-forge
curl	7.76.0	h979ede3_0	conda-forge
fontconfig	2.13.1	hba837de_1005	conda-forge
freetype	2.10.4	h0708190_1	conda-forge
fribidi	1.0.10	h36c2ea0_0	conda-forge
gcc_impl_linux-64	9.3.0	h70c0ae5_18	conda-forge
gcc_linux-64	9.3.0	hf25ea35_30	conda-forge
gettext	0.19.8.1	h0b5b191_1005	conda-forge
gfortran_impl_linux-64	9.3.0	hc4a2995_18	conda-forge
gfortran_linux-64	9.3.0	hdc58fab_30	conda-forge
graphite2	1.3.13	h58526e2_1001	conda-forge

gsl	2.6	he838d99_2	conda-forge
gxx_impl_linux-64	9.3.0	hd87eabc_18	conda-forge
gxx_linux-64	9.3.0	h3fbe746_30	conda-forge
harfbuzz	2.8.0	h83ec7ef_1	conda-forge
htslib	1.12	h9093b5e_1	bioconda
icu	68.1	h58526e2_0	conda-forge
jemalloc	5.2.1	h9c3ff4c_5	conda-forge
jpeg	9d	h36c2ea0_0	conda-forge
k8	0.2.5	h9a82719_1	bioconda
kernel-headers_linux-64	2.6.32	h77966d4_13	conda-forge
krb5	1.17.2	h926e7f8_0	conda-forge
ld_impl_linux-64	2.35.1	hea4e1c9_2	conda-forge
libblas	3.9.0	8_openblas	conda-forge
libcblas	3.9.0	8_openblas	conda-forge
libcurl	7.76.0	hc4aaa36_0	conda-forge
libdeflate	1.7	h7f98852_5	conda-forge
libedit	3.1.20191231	he28a2e2_2	conda-forge
libev	4.33	h516909a_1	conda-forge
libffi	3.3	h58526e2_2	conda-forge
libgcc-devel_linux-64	9.3.0	h7864c58_18	conda-forge
libgcc-ng	9.3.0	h2828fa1_18	conda-forge
libgfortran-ng	9.3.0	hff62375_18	conda-forge
libgfortran5	9.3.0	hff62375_18	conda-forge
libglib	2.68.1	h3e27bee_0	conda-forge
libgomp	9.3.0	h2828fa1_18	conda-forge
libiconv	1.16	h516909a_0	conda-forge
liblapack	3.9.0	8_openblas	conda-forge
libnghttp2	1.43.0	h812cca2_0	conda-forge
libopenblas	0.3.12	pthreads_h4812303_1	conda-forge
libpng	1.6.37	h21135ba_2	conda-forge
libssh2	1.9.0	ha56f1ee_6	conda-forge
libstdcxx-devel_linux-64	9.3.0	hb016644_18	conda-forge
libstdcxx-ng	9.3.0	h6de172a_18	conda-forge
libtiff	4.2.0	hdc55705_0	conda-forge
libuuid	2.32.1	h7f98852_1000	conda-forge
libwebp-base	1.2.0	h7f98852_2	conda-forge
libxcb	1.13	h7f98852_1003	conda-forge
libxml2	2.9.10	h72842e0_3	conda-forge
lz4-c	1.9.3	h9c3ff4c_0	conda-forge
make	4.3	hd18ef5c_1	conda-forge
minimap2	2.18	h5bf99c6_0	bioconda
ncurses	6.2	h58526e2_4	conda-forge
numpy	1.20.2	py39hdbf815f_0	conda-forge
openssl	1.1.1k	h7f98852_0	conda-forge
pandas	1.2.3	py39hde0f152_0	conda-forge
pango	1.48.4	hb8ff022_0	conda-forge
pcre	8.44	he1b5a44_0	conda-forge
pcre2	10.36	h032f7d1_1	conda-forge
pip	21.0.1	pyhd8ed1ab_0	conda-forge
pixman	0.40.0	h36c2ea0_0	conda-forge
pthread-stubs	0.4	h36c2ea0_1001	conda-forge
pysam	0.16.0.1	py39h051187c_3	bioconda
python	3.9.2	hffdb5ce_0_cpython	conda-forge
python-dateutil	2.8.1	py_0	conda-forge

python_abi	3.9	1_cp39	conda-forge
pytz	2021.1	pyhd8ed1ab_0	conda-forge
r-askpass	1.1	r40hcdcec82_2	conda-forge
r-assertthat	0.2.1	r40h6115d3f_2	conda-forge
r-backports	1.2.1	r40hcfec24a_0	conda-forge
r-base	4.0.3	h349a78a_8	conda-forge
r-bh	1.75.0_0	r40hc72bb7e_0	conda-forge
r-bit	4.0.4	r40hcdcec82_0	conda-forge
r-bit64	4.0.5	r40hcdcec82_0	conda-forge
r-bitops	1.0_6	r40hcdcec82_1004	conda-forge
r-blob	1.2.1	r40h6115d3f_1	conda-forge
r-brio	1.1.1	r40hcfec24a_0	conda-forge
r-cachem	1.0.4	r40hcfec24a_0	conda-forge
r-callr	3.6.0	r40hc72bb7e_0	conda-forge
r-cli	2.4.0	r40hc72bb7e_0	conda-forge
r-colorspace	2.0_0	r40h9e2df91_0	conda-forge
r-crayon	1.4.1	r40hc72bb7e_0	conda-forge
r-curl	4.3	r40hcfec24a_1	conda-forge
r-dbi	1.1.1	r40hc72bb7e_0	conda-forge
r-dbplyr	2.1.1	r40hc72bb7e_0	conda-forge
r-desc	1.3.0	r40hc72bb7e_0	conda-forge
r-diffobj	0.3.3	r40hcfec24a_0	conda-forge
r-digest	0.6.27	r40h1b71b39_0	conda-forge
r-dplyr	1.0.5	r40h03ef668_0	conda-forge
r-ellipsis	0.3.1	r40hcdcec82_0	conda-forge
r-evaluate	0.14	r40h6115d3f_2	conda-forge
r-fansi	0.4.2	r40hcfec24a_0	conda-forge
r-farver	2.1.0	r40h03ef668_0	conda-forge
r-fastmap	1.1.0	r40h03ef668_0	conda-forge
r-formatr	1.8	r40hc72bb7e_0	conda-forge
r-futile.logger	1.4.3	r40h6115d3f_1003	conda-forge
r-futile.options	1.0.1	r40h6115d3f_1002	conda-forge
r-generics	0.1.0	r40hc72bb7e_0	conda-forge
r-ggplot2	3.3.3	r40hc72bb7e_0	conda-forge
r-glue	1.4.2	r40hcfec24a_0	conda-forge
r-gtable	0.3.0	r40h6115d3f_3	conda-forge
r-hms	1.0.0	r40hc72bb7e_0	conda-forge
r-httr	1.4.2	r40h6115d3f_0	conda-forge
r-hwriter	1.3.2	r40h6115d3f_1003	conda-forge
r-isoband	0.2.4	r40h03ef668_0	conda-forge
r-jsonlite	1.7.2	r40hcfec24a_0	conda-forge
r-labeling	0.4.2	r40h142f84f_0	conda-forge
r-lambda.r	1.2.4	r40h6115d3f_1	conda-forge
r-lattice	0.20_41	r40hcfec24a_3	conda-forge
r-lifecycle	1.0.0	r40hc72bb7e_0	conda-forge
r-locfit	1.5_9.4	r40hcdcec82_1	conda-forge
r-magrittr	2.0.1	r40hcfec24a_1	conda-forge
r-mass	7.3_53.1	r40hcfec24a_0	conda-forge
r-matrix	1.3_2	r40he454529_0	conda-forge
r-matrixstats	0.58.0	r40hcfec24a_0	conda-forge
r-memoise	2.0.0	r40hc72bb7e_0	conda-forge
r-mgcv	1.8_34	r40he454529_0	conda-forge
r-mime	0.10	r40hcfec24a_0	conda-forge
r-munsell	0.5.0	r40h6115d3f_1003	conda-forge

r-nlme	3.1_152	r40h859d828_0	conda-forge
r-openssl	1.4.3	r40he5c4762_0	conda-forge
r-pillar	1.5.1	r40hc72bb7e_0	conda-forge
r-pkgconfig	2.0.3	r40h6115d3f_1	conda-forge
r-pkgload	1.2.1	r40h03ef668_0	conda-forge
r-plogr	0.2.0	r40h6115d3f_1003	conda-forge
r-plyr	1.8.6	r40h0357c0b_1	conda-forge
r-praise	1.0.0	r40h6115d3f_1004	conda-forge
r-prettyunits	1.1.1	r40h6115d3f_1	conda-forge
r-processx	3.5.1	r40hcfec24a_0	conda-forge
r-progress	1.2.2	r40h6115d3f_2	conda-forge
r-ps	1.6.0	r40hcfec24a_0	conda-forge
r-purrr	0.3.4	r40hcdcec82_1	conda-forge
r-r6	2.5.0	r40hc72bb7e_0	conda-forge
r-rappdirs	0.3.3	r40hcfec24a_0	conda-forge
r-rcolorbrewer	1.1_2	r40h6115d3f_1003	conda-forge
r-rcpp	1.0.6	r40h03ef668_0	conda-forge
r-rcpparmadillo	0.10.2.2.0	r40h306847c_0	conda-forge
r-rcurl	1.98_1.3	r40hcfec24a_0	conda-forge
r-rematch2	2.1.2	r40h6115d3f_1	conda-forge
r-reshape2	1.4.4	r40h0357c0b_1	conda-forge
r-rlang	0.4.10	r40hcfec24a_0	conda-forge
r-rprojroot	2.0.2	r40hc72bb7e_0	conda-forge
r-rsqlite	2.2.5	r40h03ef668_0	conda-forge
r-rstudioapi	0.13	r40hc72bb7e_0	conda-forge
r-scales	1.1.1	r40h6115d3f_0	conda-forge
r-snow	0.4_3	r40h6115d3f_1002	conda-forge
r-statmod	1.4.35	r40h86c2bf4_1	conda-forge
r-stringi	1.5.3	r40hcabe038_1	conda-forge
r-stringr	1.4.0	r40h6115d3f_2	conda-forge
r-survival	3.2_10	r40hcfec24a_0	conda-forge
r-sys	3.4	r40hcdcec82_0	conda-forge
r-testthat	3.0.2	r40h03ef668_0	conda-forge
r-tibble	3.1.0	r40hcfec24a_1	conda-forge
r-tidyr	1.1.3	r40h03ef668_0	conda-forge
r-tidyselect	1.1.0	r40h6115d3f_0	conda-forge
r-utf8	1.2.1	r40hcfec24a_0	conda-forge
r-vctrs	0.3.7	r40hcfec24a_0	conda-forge
r-viridislite	0.3.0	r40h6115d3f_1003	conda-forge
r-waldo	0.2.5	r40hc72bb7e_0	conda-forge
r-withr	2.4.1	r40hc72bb7e_0	conda-forge
r-xml	3.99_0.6	r40hcfec24a_0	conda-forge
r-xml2	1.3.2	r40h0357c0b_1	conda-forge
r-xtable	1.8_4	r40h6115d3f_3	conda-forge
r-zeallot	0.1.0	r40h6115d3f_1002	conda-forge
readline	8.0	he28a2e2_2	conda-forge
salmon	1.4.0	h84f40af_1	bioconda
samtools	1.12	h9aed4be_1	bioconda
sed	4.8	he412f7d_0	conda-forge
setuptools	49.6.0	py39hf3d152e_3	conda-forge
six	1.15.0	pyh9f0ad1d_0	conda-forge
sqlite	3.35.4	h74cdb3f_0	conda-forge
sysroot_linux-64	2.12	h77966d4_13	conda-forge
tbb	2020.2	h4bd325d_4	conda-forge

tk	8.6.10	h21135ba_1	conda-forge
tktable	2.10	hb7b940f_3	conda-forge
tzdata	2021a	he74cb21_0	conda-forge
wheel	0.36.2	pyhd3deb0d_0	conda-forge
xorg-kbproto	1.0.7	h7f98852_1002	conda-forge
xorg-libice	1.0.10	h7f98852_0	conda-forge
xorg-libsm	1.2.3	hd9c2040_1000	conda-forge
xorg-libx11	1.7.0	h7f98852_0	conda-forge
xorg-libxau	1.0.9	h7f98852_0	conda-forge
xorg-libxdmcp	1.1.3	h7f98852_0	conda-forge
xorg-libxext	1.3.4	h7f98852_1	conda-forge
xorg-libxrender	0.9.10	h7f98852_1003	conda-forge
xorg-libxt	1.2.1	h7f98852_2	conda-forge
xorg-renderproto	0.11.1	h7f98852_1002	conda-forge
xorg-xextproto	7.3.0	h7f98852_1002	conda-forge
xorg-xproto	7.0.31	h7f98852_1007	conda-forge
xz	5.2.5	h516909a_1	conda-forge
zlib	1.2.11	h516909a_1010	conda-forge
zstd	1.4.9	ha95c52a_0	conda-forge
